# Personalised treatment for cancer: role of biomarkers

**DOI:** 10.1186/1878-5085-5-S1-A23

**Published:** 2014-02-11

**Authors:** Michael J Duffy

**Affiliations:** 1 Clinical Research Centre and UCD School of Medicine and Medical Science, Conway Institute, University College Dublin, Dublin 4, Ireland; 2 Clinical Research Centre, St Vincent’s University Hospital, Dublin 4, Ireland

## 

Recent molecular analysis has shown that enormous molecular differences exist between cancer of particular organ, i.e., cancer exhibit major inter tumor heterogeneity. The implications of this heterogeneity are that cancers of a particular type vary widely with respect to outcome and response to therapy. In order to optimally treat cancer, it is therefore necessary to use a personalised approach [1]. This means giving the most appropriate treatment to each patient.

In order to progress to personalised treatment for cancer, a variety of different validated biomarkers are necessary, especially prognostic and predictive biomarkers. Prognostic biomarkers help predict whether a tumor is likely to be indolent and thus be cured by surgery or aggressive with the potential to metastasis and thus give rise to a recurrence. Prognostic biomarkers thus help doctors minimise overtreatment of indolent tumors and undertreatment of aggressive tumors. Currently, only a small number of biomarkers are validated for clinical use. These include uPA and PAI-1 in breast cancer, AFP, HCG and LDH in testicular germ cell cancers, microsatellite instability status in colorectal cancer and PSA in prostate cancer [1].

In contrast to prognostic biomarkers, predictive biomarkers help to identify patients who are likely to benefit of be resistant to specific therapies. An ability to upfront select the patients likely to benefit from a specific treatment can save patients from unnecessary side effects and allow them to receive therapy that is more likely to be of value [3]. Furthermore, having accurate predictive markers should also lead to major cost savings, as drugs would only be given to those patients likely to derive benefit. This cost saving, is especially important for the new biological or targeted therapies, as many of these agents are relatively expensive and have efficacy in only a minority of patients with a specific malignancy.

Amongst the best validated predictive biomarkers are the estrogen and progesterone receptors for the identification of endocrine-sensitive breast cancer, HER2 for selecting response to anti-HER2 therapies (Herceptin, lapatinib, pertuzumab and T-DM1) in breast cancer, K-RAS mutations for the selection of patients with advanced colorectal cancer who are unlikely to benefit from anti-EGFR antibodies (cetuximab, panitumumab), EGFR mutations for selecting patients with advanced non-small cell lung cancer for treatment with tyrosine kinase inhibitors (gefitinib, erlotinib), BRAF mutations for selecting patients with advanced melanoma for treatment with anti-BRAF drugs (vemurafenib, dabrafenib) and ALK translocations for identifying patients with non-small cell lung cancer likely to benefit from crizotinib [3].

Currently, the main obstacle to progress in personalised treatment for cancer is the lack of validated prognostic and predictive biomarkers. However, with the continuing decline in cost, the use of DNA sequencing of newly diagnosed tumors is likely to be one of the key technologies in making personalised treatment available for a greater proportion of patients.
